# Characterization of Miconazole Effects on Mice Fetus Liver Tissue Using FTIR-MSP

**Published:** 2017

**Authors:** Azadeh Ashtarinezhad, Ataollah Panahyab, Baharak Mohamadzadehasl, Farshad H. Shirazi

**Affiliations:** a *Department of Occupational Health, faculty of Public Health, Iran University of Medical Sciences, Tehran, Iran. *; b *Young Researchers and Elite Club, Central Tehran Branch, Islamic Azad University,Tehran, Iran.*; c *Department of Pharmacology/Toxicology, Faculty of Pharmacy, University of Shahid Beheshti/ Medical Sciences, Tehran, Iran. *; d *Pharmaceutical Sciences Research Center, ShahidBeheshti University of Medical Sciences, Tehran, Iran.*

**Keywords:** FTIR-Microspectroscopy, Teratogenic, Mice fetus liver tissue, Miconazole

## Abstract

Azole agents especially Miconazole are widely used even during pregnancy as antifungal agents. The aim of this study was to assess the usefulness of FTIR Micro-Spectroscopy for discriminating of Miconazole treated liver tissue from control liver tissue. The mice were injected with Miconazole (60 mg/Kg) on gestation day 9 and they were dissected on pregnancy day 15. The fetus fixed, dehydrated, and embedded in paraffin. Sections of liver (10 μM) were prepared from control and treated fetus groups by Microtome and deparaffinized with xylene. The spectra were collected using FTIR-MSP in the region of 4000-400 cm^-1^. All spectra were normalized to amide II band (1454 cm^-1^) after baseline correction of entire spectrum. The results were shown by 2nd derivatization of spectra and also subtracting from control spectra. Miconazole induces some minor changes in the mouse fetus liver at cellular levels when mother is exposed. The most important calculated alterations are in the production of fetus liver proteins. α helical and β sheet structures have shown significant variations, indicating protein alterations configurationally.

## Introduction

Azole agents (triazole and imidazole derivatives) are widely used against superficial and deep mycosis for oral topical, vaginal, or systemic treatment of candidiasis and coccidioidal for cryptococcal meningitis (1) even during pregnancy. Miconazole (MIC), a strong antifungal and antibacterial agent against gram- positive bacteria such as Staphylococcus aureus (2), is an imidazole antifungal agent which is occasionally used intravenously to treat severe systemic mycosis infections, while local preparations are used for topical treatment of vulvo-vaginal candidiasis or superficial skin infections caused by the dermatophytes and candida species. Pregnant women also use Miconazole though it is labeled as category C (FDA, 1980) (2-3).The effect of Miconazole in dogs has been demonstrated (2).Co-administration of Miconazole, ketoconazole, and fluconazole with acetazolamide to pregnant mice increase the frequency of forelimb ectrodactyly compared to acetazolamide alone (4). Metronidazole and Miconazole concomitant during the pregnancy especially at organogenesis period caused polydactylyl and syndactylyl in the upper organs, skeletal defects, cleft palate, the rib deformity, cranial deformity, dilatation of the renal pelvis, and bladder (5-13). Miconazole at high concentrations exhibits bactericidal activity by producing necrotic changes in cells (14). There are controversial studies for Miconazole adverse effects on animal fetus (teratogenic or embryocidal) with no controlled studies in human (2). 

**Figure 1.**
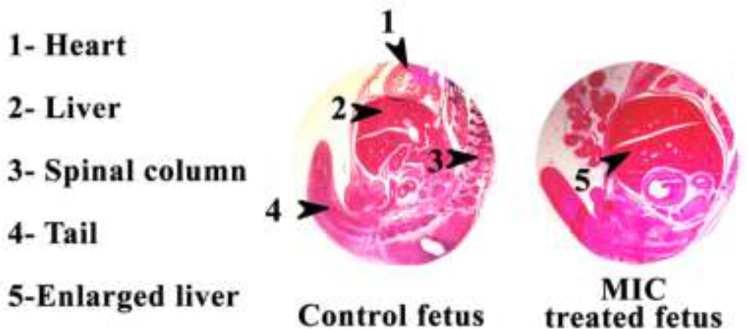
H & E stained sections of the Control and MIC treated mice fetus tissue. MIC; Miconazole

**Figure 2:**
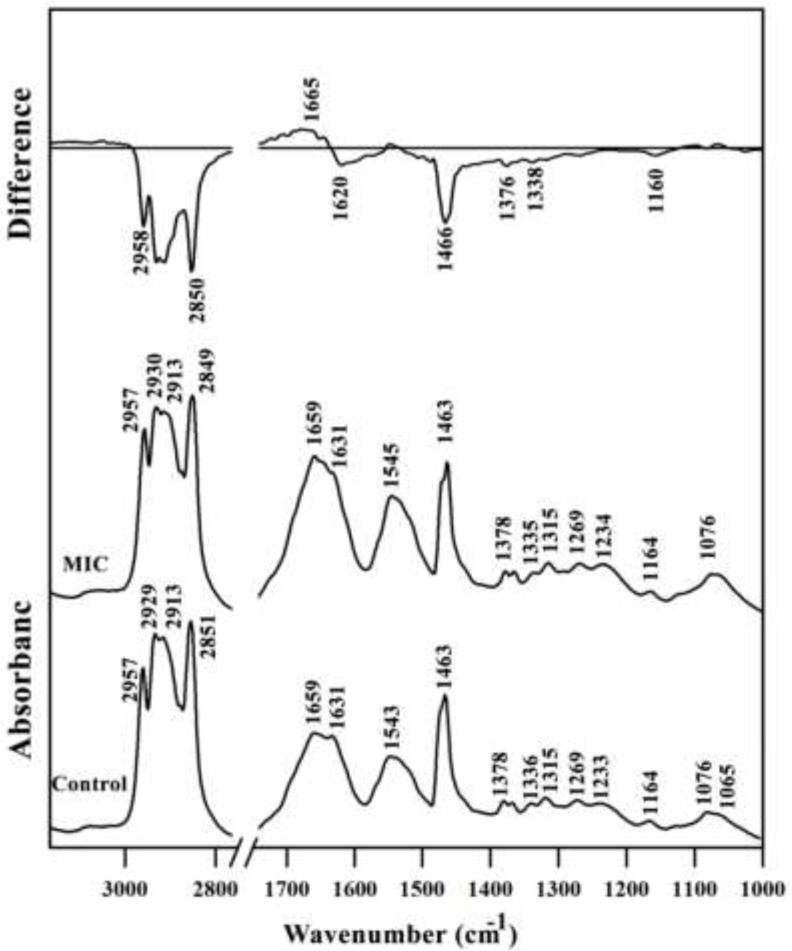
Mid-infrared spectra of Control and MIC treated mouse fetus liver tissues. The differential FTIR spectra of MIC treated liver spectra minus the spectra of control liver tissues in the 3500–1000 cm^-1^ wave number region is also presented on the top. The spectra are baseline-corrected and normalized to the amide II band. MIC; Miconazole

**Figure 3:**
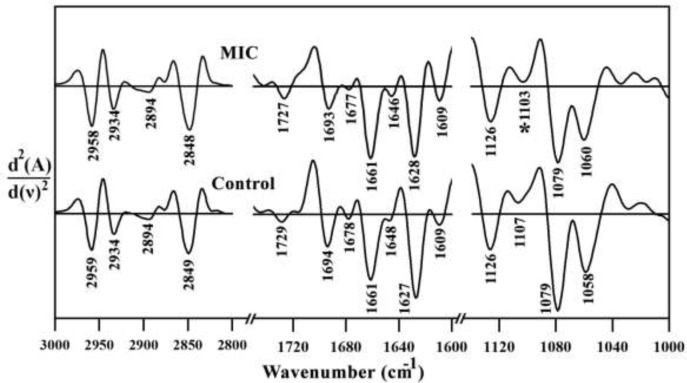
2nd derivative of mean FTIR spectra of Control and MIC treated mouse fetus liver tissues in the 3000–1000 cm^-1^ wavenumber region. MIC: Miconazole

**Figure 4:**
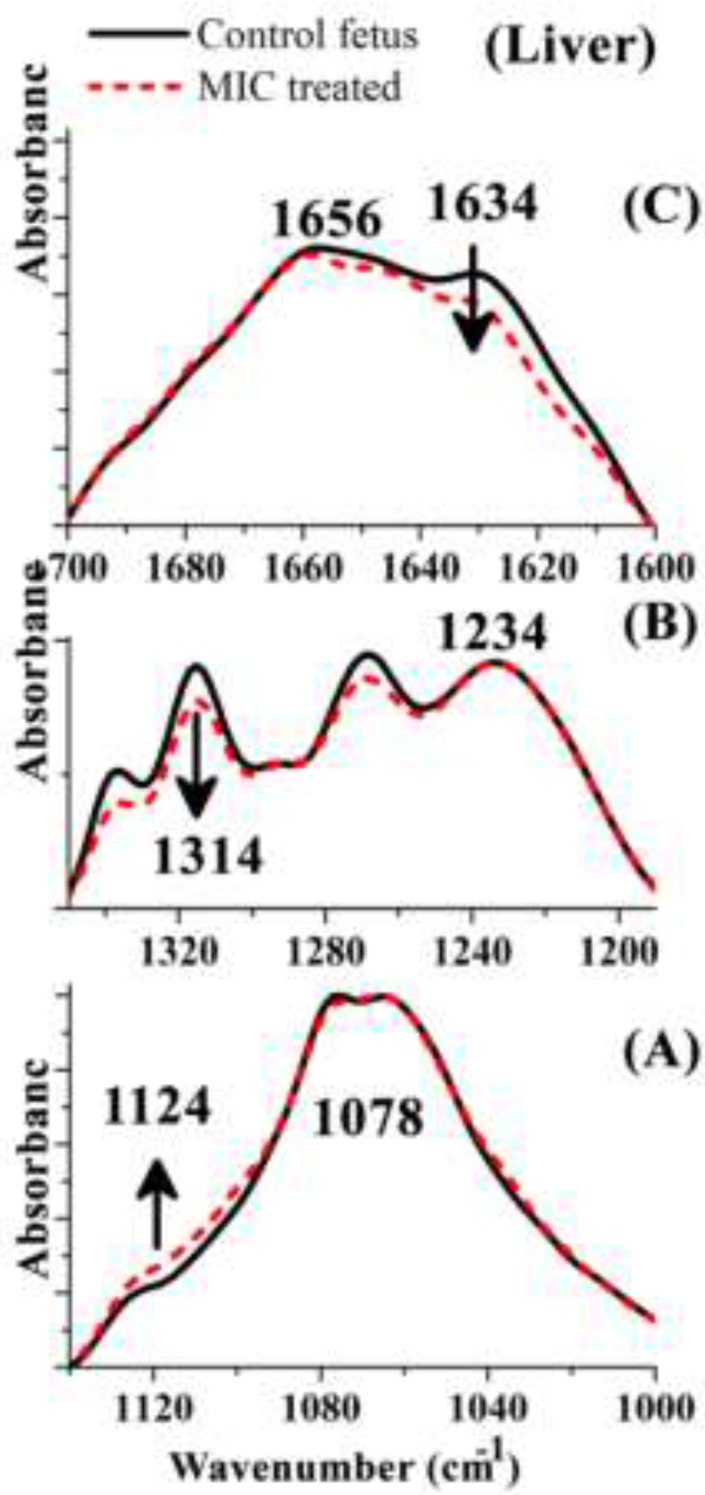
FTIR spectra of Control and MIC treated mouse fetus liver tissue in the various regions: 1000 to 1150 cm−1 (A), 1355 to1390 cm−1 (B), 1600 to 1700 cm−1 (C) and 1600 to 3000 cm−1 (D). The spectra are baseline-corrected and normalized. MIC: Miconazole

**Figure 5:**
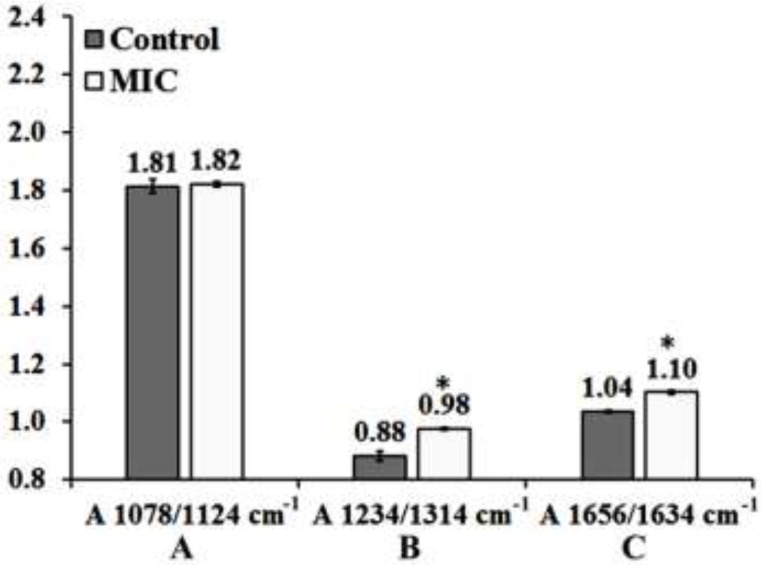
Effect of MIC exposure on the ratios of A) A1087cm^−1^/A1124cm^−1^, B) A1234cm^−1^/A1314cm^−1^ and C) A1658cm^−1^/A1630cm^−1^in the mouse fetus liver tissue. Results are expressed as Means Plot (SD as Error). Data were analyzed by one-way ANOVA test (p < 0.05). *: Significant difference in comparison to Control mouse fetus liver tissue. Exact p values for each ratio are presented in table 1. MIC: Miconazole

**Tables 1:** The statistical values of the mentioned ratios (A; A1087cm^−1^/A1124cm^−1^, B; A1234cm^−1^/A1314 cm^−1^ and C; A1658cm^−1^/A1630 cm^−1^) for control and MIC treated mouse fetus liver tissue. MIC: Miconazole

**Ratio**	**Control fetus**	**MIC treated fetus**	***F***	***P-value***
	**Mean ± SD**			
A1087/A1124 cm^-1^ RNA / Total nucleic acid content	1.81 ± 0.02	1.82 ± 0.01	0.07	0.80
A1236/A1314 cm^-1^ nucleic acid / total protein	1.12 ± 0.05	1.77 ± 0.20	24.18	1.7 ×10^-5^
A1658/A1630 cm^-1^ α-helix to β-sheet structure of total proteins	1.09 ± 0.03	1.49 ± 0.05	58.55	3.28×10^-9^

Spectroscopic techniques are used by researchers at basic and clinical levels in biology to detect malignancy, cancer, and the other disorders (15). This technique provides information for the identification and chemical distribution of cellular molecules and tissue components by the use of spectra (16-21). A series of investigations have been started to evaluate the applicability of FTIR spectroscopy for the determination of different agent’s teratogenicity such as Phenobarbital and Metronidazole effects on mice fetus liver and brain tissues (22,23). The aim of the present study was to assess the usefulness of FTIR Micro-Spectroscopy for discrimination of Miconazole treated mice liver tissue from control mice.

## Experimental

*Materials & Method*

*Sample preparation for FTIR microspectroscopy*

Miconazole was purchased from Sigma. Adult mouse (10-12 weeks) weighting 20 g were obtained from Razi institute, Iran. The mouse was fed with a standard diet with water and libitum, and kept in a room with controlled light (12:12, dark: light), temperature (22 ± 10 ºC), relative humidity (40-50%), and ventilation (15 air changes per h). They were allowed to adapt to their environment for 1 week prior to the experiments. Mice were randomly mated and pregnancies were assessed by the formation of vaginal plaque. Pregnant mice were divided into two groups and treated as follows: The control group (5 mice) received no medication and the tested group (5 mice) received Miconazole (60 mg/Kg) intraperitoneally on gestation day 9. Mice were sacrificed and dissected on day 15th of gestation. The fetuses fixed in Bouin’s solution for 18 h at room temperature, dehydrated in a graded ethanol series, and embedded in paraffin. Sections were cut by microtome at a thickness of 10 μM and deparaffinized with xylene (24, 25). Slices were mounted on a 1 mm thick KBr window for IR micro spectroscopy or placed on conventional glass slides for staining with haematoxylin and eosin (H&E) for the histological study of fetus abnormalities using light microscopy.

*FTIR microspectroscopy (FTIR-MSP)*

FTIR measurements were performed in the absorbance mode. WQF-510 Fourier transform spectrometer (Rayleigh Optics, China) was equipped with a KBr beam splitter equipped with a DLaTGS (Deuterated Lanthanide Triglycine Sulphate) detector and µMAX IR microscope (PIKE Technologies, USA). The spectra were scanned in the mid-IR range from 4000 to 400 cm^-1^, with a resolution of 4 cm^-1^. 100 scans were coded for each spectrum and the spectra were normalized against the background spectrum for mathematical processing.

*Data processing and statistical analysis*

Data were analyzed using Main FTOS IR software (routine software on the FTIR equipment). The spectra were recorded from several sites on mouse fetus liver, average spectrum from all spectra was computed. All spectra were baseline corrected and normalized in reference to the band at 1445 cm^-1^ (amide II peak) for uniformity (24). The differential spectra (MIC treated tissue spectra minus control spectra) were obtained in order to monitor the intensity variations on FTIR spectra. Second order derivatives were also calculated to show more details of spectral changes in specific regions (amide I band; 1600-1700 cm^-1^, lipids; 2800-3000 cm^-1^, nucleic acid and carbohydrate;1000-1200 cm^-1^). The results are expressed as Means Plot (SD as Error). The data were analyzed by one-way ANOVA test (p < 0.05) using Origin Pro (Version 8.5.1) software.

## Results and Discussion

*Morphological studies*

Figure 1 shows the H & E stained sections photomicrographs of Control and MIC treated mouse fetus tissues. Clearly MIC treated mouse fetus liver is bigger than the control mouse fetus liver.

*IR spectral characteristics of the drugs treated mouse fetus liver tissue*

A typical FTIR spectrum of Control and MIC treated mouse fetus liver tissues is shown in Figure 2. The information contained in such an IR absorption spectrum originates from all different types of biomolecules in the liver tissue, such as lipids, proteins, carbohydrates, and nucleic acids. The MIC treated liver tissues spectra had a lower intensity in the lipid regions (2800–3000 cm^-1^ and 1466 cm^-1^) compared to the control liver tissue owing to decreasing in total lipid content. The result of differential spectra for amide I region is somehow interesting; the intensity has decreased at 1620 cm^-1^ but increased at 1665 cm^-1^corresponding to conformational changes of proteins at the secondary structure level.

Second order derivatives results of three specific spectral regions are shown in Figure 3. No major spectral shifting was observed for amide I, lipid, nucleic acid and carbohydrate related bands, indicating no major changes occurred in molecular level of liver tissue after the Miconazole treatment.

*FTIR Quantitative Analysis of the Liver Tissues:*

We have analyzed FTIR spectra obtained from all Control and MIC treated mouse fetus liver tissues to find spectral biomarkers that can discriminate between control and drug treated mouse fetus. Three regions were selected to show any possible variations of biomolecules content in corresponding liver tissues. These regions were cut from the whole spectra, baseline corrected, and normalized to the highest peak in each specific region just to show the spectral changes visually (Figure 4).

The best discriminating values were obtained by deriving the intensity ratio of two vibration modes in each region; consequently, three absorbance ratios were calculated using the peaks heights measurement. The dimensionless ratio eliminates artifact, which may arise due to the baseline contribution underneath each band (2). The first region from 1000 to 1145 cm^−1^ (Figure 4-A) shows three overlapped absorbance bands at 1124 cm^-1^ arises from RNA absorbance, at 1087 cm^-1^ correspond to absorbance of the ν_s_ PO^2−^ of phosphodiesters of nucleic acids and at 1058 cm^−1^ related to the O–H stretching coupled with C–O bending of C–OH groups of carbohydrates. The ratio (A1087/A1124 cm^-1^) may be assumed as the features from RNA/ Total nucleic acid content ratio. The result of this ratio has been shown in Figure 5-A and Table 1, as the result presented there is not significant differences between Control and MIC treated tissues for this ratio. Therefore no major alternation occurred in nucleic acid content of liver tissue at the cellular level of fetus after the exposure of mother to Miconazole.

The 2nd region (from1200 to 1350 cm^−1^) presented in Figure 4-B in which plenty of overlapping vibrational modes associated with the absorbance of macromolecules such as proteins Amide III band in 1180 to 1300 cm^−1^, nucleic acids asymmetric PO_2_^-^ stretching of RNA and DNA in 1220 to 1240 cm^−1^, carbohydrates, and phospholipids CH_2_ wagging vibration of the acyl chains in 1250 cm^−1^-1400 cm^-1^ are apparent in this region. The band at 1236 cm^−1^ (asymmetric stretching PO^-^_2_ of nucleic acids) is compared to the band at 1314 cm^−1^ arising from Amide III band components of proteins (3). This region was selected to derive a biomarker with outstanding statistical characteristics: A1236/A1314 ratio, assigned nucleic acid to total protein of the liver tissue presumably by which might reflect the protein synthesis activity rate in cells; lower ratios indicating higher protein synthesis rate. The mean values of this ratio for the liver tissue are presented in Figure 5 and Table 1. As is shown, exposure has significantly decreased protein synthesis in these cells.

The 3rd region of 1600 to 1700 cm^−1 ^has been shown in Figure 4-C which is due to amide I band of proteins (highly sensitive to the conformational changes in the secondary structure). In this region the wavelength of 1650 to 1658 cm^-1 ^is associated with the presence of α-helix structure of proteins, while the β-sheet vibrations have been shown in 1620 cm^-1^ to 1638 cm^-1^. The absorbance ratio of A1658/A1630 cm^-1 ^is used here to measure the ratio of α-helix to β-sheet structure of mouse fetus liver cellular total proteins configurationally alterations after the exposure of mother to MIC (Figure 5-C). The result of this ratio reveals significant difference between control and MIC treated mouse fetus liver tissues. As a comparative result to control liver tissue, it is statistically obvious that protein configurations have changed after the exposure of fetus liver tissue to Miconazole.

## Conclusion

Teratology is a threshold phenomenon and its indication in limited instances where the level of alterations exceeds a threshold to be evident in morphology. Pharmaceuticals and environmental chemicals are among the teratogenic agents (26, 27). New methods to reflect the teratogenicity of different materials are needed to overcome the limitation of late morphologic evident for a safer use of materials in pregnant mothers. To overcome this limitation, we have tried to find a rational way for the use of biospectroscopy to evaluate the alterations in the fetus caused by a widely use azole medications in ladies; Miconazole. The fetal safety profile of azoles has not yet been fully defined, although both of *in-vivo* and *in-vitro* models have shown that all the tested azoles displayed teratogenic effects (26). However, the exact molecular alterations of these defects have not yet been explained. The production of ergosterol, the main sterol in the fungal cell membrane is inhibited by azole, thereby membrane fluidity and impairing membrane-associated enzyme activity, as well as cell growth and replication alters in affected infectious parasites. Azoles also interact adversely with cell membrane phospholipids, inhibit endogenous respiration, and inhibit morphogenetic transformation of yeasts to the mycelial form (4). The main question of teratology for the use of this medication in mothers is whether these mechanisms might affect fetus.

Biospectroscopy is the main technique used for some decades aiming to understand molecular alterations long enough before coming to the morphology changes. Researchers have used this technique in the discrimination of cancer from normal tissues at the molecular level (16-21). For teratology purposes, molecular alterations after exposure to Metronidazole and Phenobarbital drugs were assessed by using the FTIR-MSP in mice fetus brain and liver tissues (22, 23 and 28). Vibrational microspectroscopic techniques have the potential to diagnose biological samples at the molecular level, as these techniques are sensitive to the changes in the structure, composition and quantity of cellular components. To increase biospectroscopy accuracy in this article, we have double normalized data following mathematical extractions; spectral baseline correction, normalization, and derivatization have been used to extract reliable numbers for affiliated peaks of various cellular macromolecules, followed by absorbance values normalizations once based on popular peak of Amide and then purposely on protein synthesis machinery related peaks ratios. Peaks related to cellular nucleic acid and proteins have been selected for normalization purposely to elaborate the protein synthesis situation of cells after exposure to Miconazole. As is shown in the result section, Miconazole induces some rather important changes in the mouse fetus liver at cellular levels when mother is exposed. The most important calculated alterations are in the production and health of fetus liver proteins. While the DNA affiliated spectra have not shown significant differences between the control and Miconazole affected samples, but relative protein production was diminished. Moreover, when it came to the comparison of produced proteins in Miconazole affected tissues compared to the control, α helical and β sheet structures have shown significant variations, indicating protein alterations configurationally.

To the best of our knowledge these are the first steps in exploring biospectroscopy application in teratology. We have shown some indication for such a potency, but surely more investigation and complementary experiments are needed to finalize this step for extensive applications.
